# Hippopathology; or, Treatise on Diseases and Lameness of Horses

**Published:** 1834-07-01

**Authors:** 


### HIPPOPATHOLOGY; OR, SYSTEMATIC TREATISE ON THE DISORDERS AND LAMENESS OF THE HORSE, &c. &c. By *William Percivall*, M.R.C.S. Vol. I. pp. 331. Octavo. Longman's, 1834.

OF all domesticated animals, the HORSE has the most powerful claims on the sympathy and attention of the medical practitioner. The lawyer may construct his briefs—the parson may perform his clerical duties—and the merchant may grow rich in his counting-house, with little assistance from the horse; but to the medical man this spirited and intelligent animal is as essential as the knowledge which he acquires in the schools, or the medicines which he prescribes for his patients. The horse is his companion and best friend in the town and in the country. He carries him proudly through the crowded street—he conveys him safely over the midnight heath. He shares with his master in all the toils, and in many of the dangers of professional life. He is as much exposed to the elements—more indeed than the physician and surgeon—and he is not exempt from many of the diseases to which his superior is liable. To inflammation, fever, rheumatism, organic diseases of the heart, liver, and lungs, the horse is very prone; and the study of his maladies is as necessary to the medical practitioner, in a pecuniary point of view, as it is interesting in a pathological.

No one can question the competency of Mr. Percivall for the task which he has here imposed on himself, and we have no doubt that the work, when completed, will be very popular in the medical profession. It is to consist of three volumes, of which the one before us is the first. It treats especially of the external disorders of the body, including, however, several that, in human pathology, we should denominate internal, as fever, influenza, strangles, rabies, dropsy, &c.

An examination into the health of animals, and especially of the horse, is really instructive, even to the purely medical practitioner, as will probably appear, in the course of this brief review. The natural state of animals can hardly be said to be subject to disease; yet in each year many animals, and particularly the horse, are obliged to undergo certain revolutions in the system, which disturbs the health more or less. The satin coat of the Summer falls in the Autumn, preparatory to the growth of a pilous covering for the Winter. In Spring, a counter-revolution takes place, and these operations are accompanied by a certain degree of debility, predisposing to disease. Some revolution also takes place, at Spring and fall, in the human constitution, for it is at these periods, that we observe the aged and ailing more frequently nipped off than during the intervening epochs.

But it is to domestication that the horse owes almost all his evils, as it is to refinement and civilization that man owes nine-tenths of his bodily sufferings, and the doctor nine-tenths of his fees. When the horse, for instance, comes to be housed and treated with care and kindness by man, then begin his susceptibilities to those very diseases which affect his lord and master! Even the sturdy ass and stubborn mule, when domesticated, bear diseases ill, as our author had opportunities of witnessing during the Peninsular war.

In the transition from a state of Nature to a state of domestication, the horse receives most of his morbific impressions from air, exercise, grooming, and shoeing. On each of these subjects, our author makes some excellent remarks, all bearing more or less on human pathology. Thus the horse is greatly injured by the heated and impure air of the stable, in the same way as man and woman, too, are deteriorated in health, by the crowded assembly-room—the stifling theatre—and the murkey back-rooms of shops and dwelling-houses. The ventilation, draining, and cleanliness enjoined in the cavalry stables, where great numbers of horses live together, prevent sickness in a most astonishing manner, The hay and corn, though in a very different condition from the same materials in a succulent condition, do little or no injury to the horse, when the *quantity* is proper, and the circumstances under which the food is taken are favourable. The following remark is interesting.

"The only drink the horse will take, either in or out of the stable, is *water*. All that we have to guard against is, its being given at improper times, and in improper quantities. In a general way, drink is given too sparingly: after work is done, and while the body is cool, there can be no good reason for stinting the animal of this wholesome beverage. In a state of nature he helps himself to water; and from that circumstance, as well as the one of his food being green and succulent, drinks much less than when in the stable. I once made an experiment with two horses who had (leaden) mangers for containing their water, by the side of those that held their corn; and I found that when these mangers were filled, and the animals were allowed to drink *ad libitum*, they invariably consumed less fluid than when watered in the ordinary way." 5.

The theories that have been broached respecting man's drink are mostly erroneous. When thirst occurs, there can be no impropriety in slaking it—even in the midst of our meals—Abernethy to the contrary notwithstanding.

In respect to exercise, our author observes that it is difficult to say which is the more injurious of the two extremes—absolute rest, and inordinate labour. It is just the same in man. The unexercised female, in high life, is subject to as many diseases as the poor girl in the manufactory, working 14 or 15 hours in the day!

As to shoes, though we believe that man was destined to go bare-footed—or at the most with sandals; yet we allow that, with all the misery of dancing-pumps and dandy boots, the poor horse is worse off than his master.

The interference of the farrier is the greatest evil which the horse has to dread! Yet it is a necessary evil, after he has left his pasture-grounds, and taken up his abode in town or city, even with all the advantages of macadamized streets.

On the transition from health to disease in the horse, our author makes many judicious observations. Among the causes of this transition in the horse as in man, PLETHORA is conspicuous. More food is taken by both these animals than is necessary for the nutrition of the body, or proportionate to the exercise thereof. Defective or inadequate secretion and excretion result from this plethora, and tend, in return, to augment it. The congestion consequent on plethora, in the horse, most frequently shews itself in the lungs—probably from the circumstance of horses being put to quick motion directly after long quietude and good feeding in the stable. They often suffer, like man, in the brain, from this plethoric congestion, hence, "it happens every now and then that a horse dies suddenly, in a fit of vertigo or staggers, without having manifested any signs whatever of previous indisposition."

"The *blood* in sheep, a disorder to which the French give the name of *maladie de sang*, is the effect of plethora and cerebral congestion, induced by luxuriant pasture and other causes similar to the above. All on a sudden, the sheep—up to that moment in apparent health—stands aghast in the midst of the others, staggers and falls prostrate: his breathing becomes laborious; he foams at the mouth, and gasps with it wide open; he rattles in his throat; blood issues from his nose, and even from his anus: he becomes convulsed and dies." 17.

These facts may serve as a lesson to man!

The heart, as might be expected from the spirited exertions of the horse, is often affected by dilatation or hypertrophy—and auscultation is here an important knowledge, and one which may be of some pecuniary value to those who purchase horses. We had an opportunity once of examining a horse that appeared in excellent condition; but on applying the ear to the animal's side, we discovered evident symptoms of disease of the heart. We afterwards learnt that this horse was easily put out of breath on any considerable exertion.

The liver of the horse is very subject to congestion.

"In old horses that are very fat, and whose work is but occasional or trifling it is not a very uncommon accident to have the gland burst from congestion; in which case it displays a mass of seemingly nothing else but congealed black blood." 18.

The mucous membrane of the trachea and bronchi is peculiarly susceptible of inflammation—hence bronchitis is one of the common diseases of the horse. But of this more hereafter. Passing over some curious and interesting observations, we find, under the head of "signs of disease" in the horse, some remarkable analogies with those signs which denote a deviation from health in the human subject. The horse is "off his feed,"—an expression which many a biped patient has complained to us of, in the course of our practice! His spirits fail—he "hangs heavy on hand, at his work or exercise,"—and the number of the pulse is augmented. The surface of the body loses its natural smoothness, gloss and general warmth—the coat stares, and the ears and extremities are chilled. Much may be gathered from the state of the respiration. The "heaving or panting of the flanks," corresponds with that abdominal or diaphragmatic respiration in man, which augurs something wrong about the chest, preventing free thoracic expansion.

Were we to indulge our own inclinations, we would run our parallel between disease in this useful quadruped, and those of god-like man, to a great length; but we dare not, lest we should be accused of derogating from the dignity of the Lords of the Creation. We shall, therefore, only allude to one more subject, and close our notice of this volume.

### Fever.

It appears that in veterinary medicine there have been Broussaians as well as among ourselves.

"The old works on farriery present us with copious accounts of fevers; the veterinary school in this country set out with denying their existence. I commenced practice myself with a belief that there was no such disease in horses as abstract fever; but I soon found myself compelled to alter my opinion: at least I met with diseases which I was unable to refer to any other source or heading. And I believe I may add, that at the present day, the revival of this doctrine is pretty general amongst veterinarians." 142.

After alluding to the definition and short description of fever given by Cullen and Fordyce; the latter of whom considers continued fever as a succession of ague fits, with slight remissions instead of intermissions. Professor Coleman remarks, "that the horse is not subject to fever." The present author thinks that the animal in question certainly is not subject to *this* kind of fever—but, at the same time avers that the horse is liable to general fever, without reference to any appreciable local affection.

"That heat of body and accelerated pulse, together with the usual functional disorder attendant thereon, are to be found as well, and (making allowances for the number of their diseases) as often in horses as in men, no one will attempt to dispute: it only remains, therefore, to inquire, if these febrile symptoms in all cases admit of being referred to local or organic disease. Will any body take on himself to say, that he never met with these symptoms but in connexion with manifest disorder of some one or other organ or part of the body in particular? I think not. On the contrary, every one must confess, that, if such constitute fever, there can be no doubt of its existence in horses; and that, so far from its being a rare or disputable case, it is one of constant daily occurrence." 144.

Fever in horses, as in man, is generally ushered in by coldness of more or less intensity—sometimes even by a rigor, succeeded by heat and reaction, with the principal phenomena observed in the human race. The causes are generally alternations of temperature—over-exertion—and too high feeding. Idiopathic fever in the horse is seldom dangerous where no other disease coexists. It is cured by depletion.

But we must close our notice of this work, by strongly recommending it to our brethren, few of whom can afford to live *without a horse.* Nothing can put in a clearer point of view the march of intellect than the contrast between the old books on farriery and the modern works on veterinary medicine. The farrago of receipts and nostrums is now changed into accurate and scientific anatomy and physiology; while the therapeutics of the stable are reduced to even greater simplicity than in the wards of an hospital. To such perfection, indeed, has veterinary medicine arrived, that we had rather trust our body in the hands of a modern horse-doctor than in those of an ancient physician. An Hippocrates and a Sydenham were excellent observers of diseases, for they watched them throughout all their phases of increment and decline, with little interference on their parts. A Coleman and a Percivall can also observe accurately the trains of morbid phenomena in horses, aided by the lights of human and comparative anatomy and physiology, while they can apply the most energetic and successful remedies.

